# Reporting treatment planning, delivery, and dose evaluation for 3‐dimensional MLC‐based SFRT: A single institutional clinical experience

**DOI:** 10.1002/acm2.70879

**Published:** 2026-10-26

**Authors:** Joshua Misa, Jovan Pierre‐Charles, William St. Clair, Damodar Pokhrel

**Affiliations:** ^1^ Department of Radiation Medicine, Medical Physics Graduate Program University of Kentucky Lexington Kentucky USA; ^2^ Department of Radiation Medicine Indiana University School of Medicine Indianapolis Indiana USA

**Keywords:** crossfire, logistic regression, outcome modeling, outcomes, overall survival, SFRT, TCP

## Abstract

**Purpose/Objective:**

We report our institutional clinical experience of treatment planning, dose evaluation, and delivery via the same‐day 3D MLC‐based crossfire method for spatially fractionated radiotherapy (SFRT) in conjunction with combination therapy. Additionally, we aimed to model our clinical outcome data to provide clinical insights into treatment planning using this SFRT method for unresectable bulky tumors.

**Materials/Methods:**

A total of 137 large tumors in 131 patients with different histopathologies were treated with the 3D MLC‐based SFRT method using MLC‐fitting for a 1 cm cylindrical high‐dose and a 2 cm center‐to‐center distance at the isocenter. Physical and biological effective dose metrics for the SFRT target were reported, as were the maximum EQD2 to nearby critical organs. Clinical follow‐up data included tumor local control evaluated on post‐treatment CT scans, pain relief, toxicity profiles, and overall survival (OS). Tumor control probability (TCP) and pain relief probability were modeled using multivariable logistic regression, while normal tissue complication probability (NTCP) was modeled using univariable logistic regression. Lastly, OS was modeled with a univariable Cox hazard model.

**Results:**

This cohort had a median tumor volume of 477.55 cc (range, 21.47–7879.87 cc). The patient cohort was predominantly male (61.8%), with most treated sites in the abdominal‐pelvic area (37.2%). Furthermore, the most common SFRT prescription was 15 Gy in 1 fraction (85.4%), and most tumors received follow‐up treatment (126/137) with palliative intent (75/126). The median SFRT dose metric values for D5%, D10%, D50%, Dmean, D90%, D95%, EUD, and PVDR are 13.2 Gy, 11.8 Gy, 6.9 Gy, 7.3 Gy, 3.5 Gy, 2.4 Gy, 5.3 Gy, and 3.4. The resulting median target BED metrics, BED5%, BEDmean, and BED95% was 71.5, 54.6, and 44.4 Gy. A TCP rate of 76.5% (75/98), and a pain relief rate of 72.0% (59/82) was found. Using multivariable logistic regression, none of these dose metrics were statistically significant with TCP or pain relief outcomes. In total, 95 SFRT patients were available for toxicity assessment, with 65 (68.4%) reporting no toxicities and 7 (7.4%) reporting grade 3+ events. Skin toxicity and xerostomia were statistically significantly correlated with maximum EQD2 values to the skin (*p* = 0.022) and parotid glands (*p* = 0.038). The Cox hazard model showed that increases in BED metrics were statistically significantly associated with longer OS; however, increases in SFRT dose metrics were not. We reported OS rates of 51.8%, 36.3%, and 30.3% at 6, 12, and 18 months, respectively. Moreover, we found differences in OS between patients who received a combination palliative treatment to those who received a curative treatment (*p* = 0.002).

**Conclusion:**

Here, we report our single‐institution results using the 3D‐MLC‐based crossfire technique for SFRT treatment and the resulting outcome models. Further refinement of this predictive model for SFRT in a larger, more diverse cohort is ongoing to validate our findings.

**Abbreviations**
AUC: Area Under the Curve
BED: Biological Equivalent Dose
CBCT: Cone‐Beam Computed‐Tomography
CI: Confidence Interval
EQD2: Equivalent Dose in 2 Gy‐Fractions
GTV: Gross Tumor Volume
HR: Hazard Ratio
NTCP: Normal Tissue Complication Probability
OS: Overall Survival
PVDR: Peak‐to‐Valley Dose Ratio
ROC: Receiver Operating Characteristic
SFRT: Spatially Fractionated Radiotherapy
TCP: Tumor Control Probability

## 1 INTRODUCTION

Spatially Fractionated Radiotherapy (SFRT) is a unique treatment technique characterized by a highly heterogeneous dose distribution and is commonly used to treat large, bulky tumors greater than 6.0 cm in diameter. This is commonly seen as an alternating pattern of high‐ and low‐dose cylindrical rods or spheres within the tumor. This SFRT technique has historically shown promising clinical outcomes in both palliative and curative settings ^1^, ^2^, ^3^, ^4^, ^5^. A retrospective study by Mohiuddin et al. ^3^, analyzing patients treated from 1995 to 1998, reported a 78% pain response rate and a 72.5% clinical benefit (complete or partial tumor response), with no grade 3 or higher radiation‐induced toxicities in the palliative setting. In the curative setting, a study by Huhn et al. ^5^ analyzed head‐and‐neck patients treated with SFRT followed by radiation therapy and found an overall tumor control rate of 92%. Historically, these treatments were delivered with a single field with a physical Cerrobend GRID block attached to the head of a LINAC. Recent advances have led to significant innovations in the planning and delivery of SFRT. Current practices for SFRT delivery include, but are not limited to, forward‐planned MLC‐based and VMAT‐based techniques, proton SFRT, and Tomotherapy ^6^, ^7^, ^8^, ^9^, ^10^, ^11^, ^12^. Despite these new implementations of SFRT, the mechanisms behind its high efficacy remain under investigation. It has been hypothesized, with strong evidence, that SFRT induces indirect cell kill through the radiation‐induced bystander effect (RIBE), the intratumor immune response, and tumor microvasculature damage ^13^, ^14^, ^15^, ^16^, ^17^, ^18^, ^19^, ^20^.

This study focuses on a 3D MLC‐based SFRT technique called the crossfire technique ^6^, ^21^, ^22^. This technique uses up to 6 gantry angles to generate high‐dose rods within the tumor. The advantage of this technique is its forward‐planning approach, which enables same‐day treatments. Additionally, because it is MLC‐based, it allows pre‐treatment setup verification and corrections via cone‐beam CT (CBCT), which was otherwise unavailable with the physical GRID block. A study by Knight et al. ^22^ demonstrated the efficacy of this technique across 23 patients, reporting a clinical benefit rate of 79.2% and pain relief in 86.9% of patients. Furthermore, they reported that 2 of 23 patients (8.7%) experienced Grade 3 toxicities, and only 1 patient (4.3%) experienced Grade 4 toxicity.

Many institutions have reported their outcomes and experiences with SFRT with varying degrees of success. However, most of these studies focus solely on the SFRT aspect, when clinically these tumors will receive a follow‐up course of radiation treatment. Additionally, there is a lack of dose reporting for both SFRT and the follow‐up courses these patients receive, which is a necessity for this technique to become standardized. Currently, there are no recommended guidelines or protocols for treating these large, bulky tumors with SFRT and follow‐up radiation, aside from recommendations on which dose metrics to report ^23^, ^24^. This study seeks to accomplish two tasks: reporting SFRT dose metrics, cumulative treatment dose metrics, as well as our institution's clinical outcome data; and, secondly, to model our clinical outcome data using the extracted dose metrics. Future research will expand to different SFRT techniques and fractionated SFRT.

## 2 MATERIALS AND METHODS

### 2.1 Patient cohort

Between October 2019 and March 2025, our institution treated 154 patients with the 3D‐conformal MLC‐fitting SFRT method. Patients without follow‐up were excluded, resulting in a cohort of 137 tumors across 131 patients. Follow‐up data included tumor control, pain relief, toxicities, and overall survival (OS). All patients underwent clinical evaluation at our center. Pain relief was considered subjective, patient‐reported alleviation of initial symptoms documented in clinical records. For patients with multiple treated sites, pain relief was assessed at each site. Objective radiographic tumor response was evaluated by comparing GTVs on CT‐simulation scans with sequential diagnostic images (CT, MRI, PET‐CT) over the follow‐up period. The RECIST (v1.1) criteria were utilized to determine tumor response as either complete response, partial response, stable disease, or progressive disease. Local tumor control was defined as tumors achieving either a complete response, a partial response, or stable disease, as observed in post‐treatment follow‐up CT simulation. Acute toxicity was graded using the Common Terminology Criteria for Adverse Events (v4.03).

### 2.2 Patient simulation, treatment planning, and delivery

Site‐specific patients were positioned supine and immobilized either with a long thermoplastic head‐and‐neck mask and their arms at their sides, or with a Vac‐Lok bag (CIVCO, Orange City, IA) and their arms above their heads. Planning CT images were acquired on a SOMATOM go Open Pro CT scanner (Siemens Healthineers, Malvern, PA) with a 512 × 512 pixel matrix and a slice thickness of 2.0 mm. The 3D CT images were then imported into the Varian Eclipse treatment planning system (TPS, Varian Medical Systems, Palo Alto, CA), where the attending physician delineated the gross tumor volume (GTV) and nearby OARs for dose reporting. The patients treated received SFRT planned with a 3D MLC‐based technique called the crossfire technique ^6^, ^22^. This technique utilizes up to 6 coplanar gantry angles spaced 60 degrees apart. The MLCs are first fitted to the GTV, then are adjusted to have 1 cm openings and 1 cm closing. This results in a plan of high‐dose cylinders, 1 cm in diameter, with a center‐to‐center spacing of 2 cm throughout the GTV. The advantage of this technique is that it requires minimal physics resources and allows for same‐day treatment if desired.

Most patients received a single dose of nominal 15 Gy SFRT to the GTV (some received 12 Gy or 20 Gy), followed by either palliative or curative intent. Our institution's SFRT prescription dose is defined as the peak dose of the SFRT dose distribution. Planning was performed for patients with palliative intent, and these patients initiated their palliative courses two to three days after receiving SFRT. For patients treated with curative intent, combination therapy with inversely optimized VMAT plans was generated, and treatment was initiated about 1 week after SFRT.

Our clinic implements a SBRT‐like procedure for treatment setup and delivery, in which patients are immobilized either using a long head and neck mask or with a Vac‐Lok bag depending on the treatment site. All SFRT and follow‐up plans had dose verification performed using an in‐house Monte Carlo program before the patient's first treatment. Additionally, patient‐specific QA measurements were performed using EPID‐based dosimetry. Our clinic uses a gamma criteria of 3%/3 mm with a low‐dose threshold of 10%, with plans requiring a gamma passing rate of 90%. Before delivery of both the SFRT and follow‐up treatment, patients are initially aligned to external skin markers using in‐room lasers. Then, a kV‐CBCT image set is acquired and co‐registered with the planning CT image set. Patient alignment corrections are then applied with the use of 6DOF PerfectPitch couch shifts.

### 2.3 Dosimetric parameters

Typically, after the SFRT plans are delivered, patients receive additional radiation treatments. The extracted dose metrics used for analysis account for the dose delivered by both the SFRT plan and follow‐up treatments. However, due to the heterogeneity of SFRT plans and differences in fractionation, a simple plan‐sum is insufficient to extract dose metrics. Because of this, we used an s‐BED method that converts plans from physical doses to biological effective doses (BED) and then sums the distributions ^25^. This method uses the linear‐quadratic model with an *α*/*β* = 10 Gy for tumor voxels and *α*/*β* = 3 Gy for normal tissue voxels. From the summed BED distribution, we extracted the following GTV metrics: BED5%, BED10%, BED50%, BED90%, BED95%, and BEDmean. Analysis of OAR doses was performed using the equivalent dose in 2 Gy fractions (EQD2) to nearby structures, as this provides a more clinically relevant value than BED. The extracted EQD2 metrics utilized for OAR analysis was the maximum EQD2 and mean EQD2 delivered to these OARs. Additionally, we separately examined the dose metrics solely from the SFRT plan. These metrics consisted of D5%, D10%, D50%, D90%, D95%, mean dose as well as peak‐to‐valley‐dose‐ratio (PVDR) defined as either, PVDR = D10% ÷ D90%. PVDR extracted was derived from DVH metrics of the tumor contour, which loses the spatial information of the dose. It should be noted that the PVDR reported in this work may not fully represent both the spatial distribution of the high and low‐dose regions as well as the potential correlation with indirect cell kill induction. Additionally, equivalent uniform dose (EUD) was calculated for the GTV using an *α*/*β* = 10 Gy. EUD is the uniform dose delivered that causes equivalent biological damage as a heterogeneous dose delivered ^26^. These metrics were used based on recommendations from the radiosurgery society (RSS) white paper and the SFRT working group ^23^, ^24^.

### 2.4 SFRT outcome modeling

Multiple models were used and implemented based on follow‐up outcome data. For tumor control probability (TCP) and pain relief, a multivariable logistic regression model was implemented. The endpoint for TCP was defined if local control was achieved or not. This was evaluated using the RECIST (v1.1) criteria, in which local control being achieved is defined as the tumor not experiencing progressive disease. A multivariable model was used to evaluate the combined effects of the dose metrics and clinical factors, including patient sex, follow‐up treatment intent, and treatment site. So all the dose metrics and clinical factors were incorporated simultaneously in the TCP and pain relief model. For both the TCP and pain relief model, bootstrap sampling was performed for internal validation. For the TCP and pain relief model, Receiver operating characteristic (ROC) curves were generated to evaluate the performance of the models. Area under the curve (AUC) was calculated to assess the model's ability to discriminate between outcomes; an AUC of 0.5 indicates no discriminative ability, and 1.0 indicates perfect discrimination. Treatment intent was determined based on whether the EQD2 of the follow‐up prescription was ≥ 40 Gy (curative intent) or < 40 Gy (palliative intent). To estimate the normal tissue complication probability (NTCP), an univariable logistic regression was used to model specific toxicities as a function of the maximum EQD2 or the mean EQD2 dose. To quantify uncertainty and improve the robustness of these models, 1000 bootstrapped samples were generated with replacement, and the model coefficients were fit for each sample. A 95% confidence interval (CI) was then derived. Additionally for internal validation, each bootstrap sample was used to refit these models, and new AUC values were calculated. The difference in these bootstrapped AUC and original AUC values was used to estimate optimisms and to obtain an optimism‐corrected estimate of model discrimination. We report both the apparent AUC from the original data set and the optimism‐corrected AUC in this study. For the OS follow‐up outcome, an univariable Cox Hazards model was used ^27^. The metrics analyzed were extracted GTV dose metrics and clinical factors, including patient sex, follow‐up treatment intent, and treatment sites. Additionally, a Kaplan‐Meier estimator was used to determine OS at 6, 12, and 18 months ^28^.

## 3 RESULTS

### 3.1 Patient characteristics

The cohort consists of 131 total patients with 137 tumors treated. Our institution has treated more male patients with SFRT compared to female patients (61.8% vs. 38.2%). Most cases where SFRT was utilized were for abdominal/pelvis tumors (37.2%), with head/neck (HN) patients trailing behind (32.8%). Across the entire cohort, the median GTV volume was 477.6 cc, and tumors ranged from 21.5 cc to 7879.9 cc. The prescribed dose for most SFRT patients was 15 Gy in 1 fraction (85.4%). Prescription doses of 12 Gy/1 fx and 20 Gy/1 fx were also used at a lower frequency. After SFRT, most tumors received follow‐up treatment (92.0%), with either curative intent (40.5%) or palliative intent (59.5%). The median prescription for the curative course of radiation was 60 Gy in 30 fractions, and for the palliative course, 30 Gy in 10 fractions. Table 1 shows a breakdown of patient characteristics from our SFRT cohort.

**TABLE 1:** Patient characteristics of the patients treated with SFRT utilizing the 3D MLC‐based Crossfire technique at our institution. Reported values are either total or median (range).

Characteristics	Statistics
No. of Treated Lesions	137
No of Patients	131
**Sex**	
Female	50 (38.2%)
Male	81 (61.8%)
**Treatment Site**	
Head/Neck	45 (32.8%)
Chest/Lung	39 (28.5%)
Abdomen/Pelvis	51 (37.2%)
Extremity	2 (1.5%)
Lesion Size (cc)	477.6 (21.5 –7879.9)
**SFRT Prescription**	
12 Gy/1fx	6 (4.4%)
15 Gy/1fx	117 (85.4%)
20 Gy/1fx	14 (10.2%)
Follow‐Up Treatment	126 (92.0%)
**Treatment Intent**	
**Curative**	51/126
Prescription (Gy)	60 (30–70)
No of Fractions	30 (5–35)
**Palliative**	75/126
Prescription (Gy)	30 (14.8–40)
No of Fractions	10 (4–20)

### 3.2 Clinical experience

Here we present two clinical cases as examples of how SFRT and follow‐up treatments are planned. The first example case shown in Figure 1 represents a clinical pelvis SFRT case with a tumor volume of 501.5 cc. This patient was prescribed 15 Gy in 1 fraction for their SFRT course and then received a follow‐up course of 37.5 Gy in 15 fractions. Their SFRT course was planned with 5 coplanar gantry angles at 30, 150, 210, 270, and 330 degrees and delivered at 10 MV. The reported D5%, D10%, D50%, Dmean, D90%, D95%, and EUD from the SFRT course were 14.11 Gy, 13.08 Gy, 7.93 Gy, 8.44 Gy, 5.00 Gy, 4.52 Gy, and 6.59 Gy, respectively. For the follow‐up treatment, 2 static beam angles (148 and 325 degrees) were used, each with a 25‐degree wedge, and delivered at an 18 MV beam. The follow‐up treatment plan achieved D100% of 99.7%. The combined treatment's BED metrics for the GTV were: 83.04 Gy (BED5%), 79.24 Gy (BED10%), 63.38 Gy (BED50%), 65.49 Gy (BEDmean), 56.36 Gy (BED90%), and 55.30 Gy (BED95%). The OARs contoured for this patient were the bowel (light blue), bladder, rectum, femoral heads (green), and the cauda equina (yellow). The reported maximum EQD2 for these OARs from the combined treatment were: 51.14 Gy (bowel), 0.41 Gy (bladder), 1.56 Gy (rectum), 2.25 Gy (femoral heads), 45.97 Gy (cauda equina).

**FIGURE 1:**
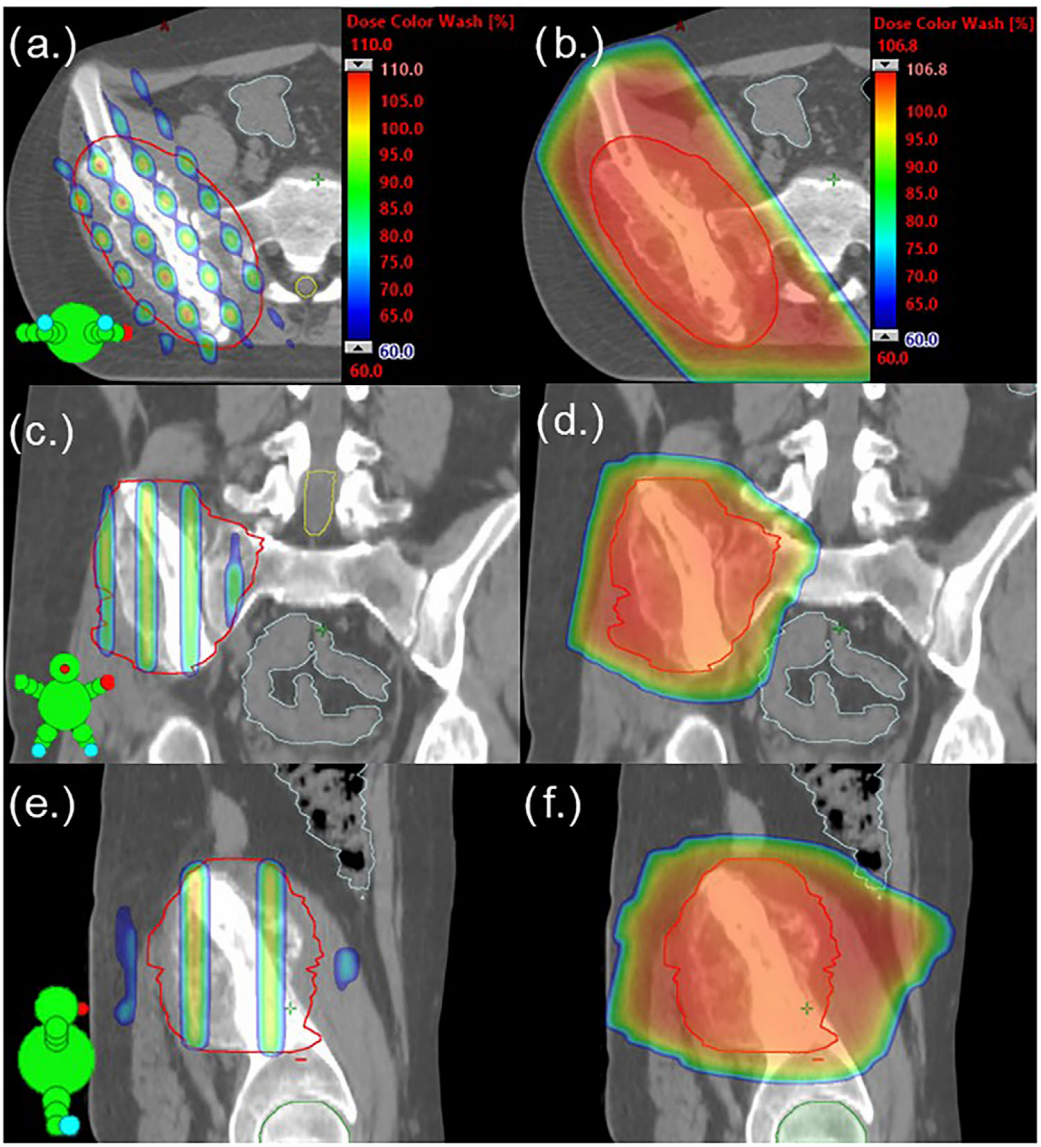
Demonstrated is a clinical right pelvis case with a large mass, prescribed with an initial course of SFRT (15 Gy/1fx) followed up with a palliative course (37.5 Gy/15fx). Shown in (a), (c), and (e) are the axial, coronal, and sagittal views of the SFRT course with an isodose color wash from 60–110% of 15 Gy. Shown in (b), (d), and (f) are the axial, coronal, and sagittal views of the follow‐up treatment course with an isodose color wash from 60–106.8% of 37.5 Gy. Shown is the contoured GTV (red), bowel (light blue), cauda equina (yellow), and the femoral heads (green).

The second example shown in Figure 2 is a clinical case of an HN tumor (429.05 cc) treated with SFRT (15 Gy/1 fx) followed by follow‐up treatment (30 Gy/5 fx). The SFRT course was planned in reverse, using 5 coplanar gantry angles at 30, 90, 150, 210, and 330 degrees, and delivered with a 6 MV beam. The reported D5%, D10%, D50%, Dmean, D90%, D95%, and EUD from the SFRT course were 12.5 Gy, 10.94 Gy, 5.83 Gy, 5.95 Gy, 0.60 Gy, 0.19 Gy, and 3.85 Gy, respectively. For the follow‐up treatment, 4 coplanar partial VMAT arcs (179–320 degrees) were used and delivered with a 6 MV FFF beam. The follow‐up plan achieved a V95% of 98.5% and a D90% of 30.83 Gy. The combined treatment's BED metrics for the GTV were: 79.51 Gy (BED5%), 74.15 Gy (BED10%), 60.85 Gy (BED50%), 61.84 Gy (BEDmean), 51.97 Gy (BED90%), and 50.60 Gy (BED95%). In Figure 2, the contoured OARs of importance are the brain (brown), brainstem (blue), left mandible (dark blue), right mandible (green), oral cavity (light blue), trachea (light brown), lips (purple), larynx (light green), right parotid (cyan) and spinal cord (yellow). The reported maximum EQD2 for these OARs from the combined treatment were: 68.3 Gy (brain), 9.27 Gy (brainstem), 73.84 Gy (left mandible), 22.41 Gy (right mandible), 40.76 Gy (oral cavity), 11.61 Gy (trachea), 16.71 Gy (lips), 23.34 Gy (larynx), 5.87 Gy (right parotid), 16.28 Gy (spinal cord).

**FIGURE 2:**
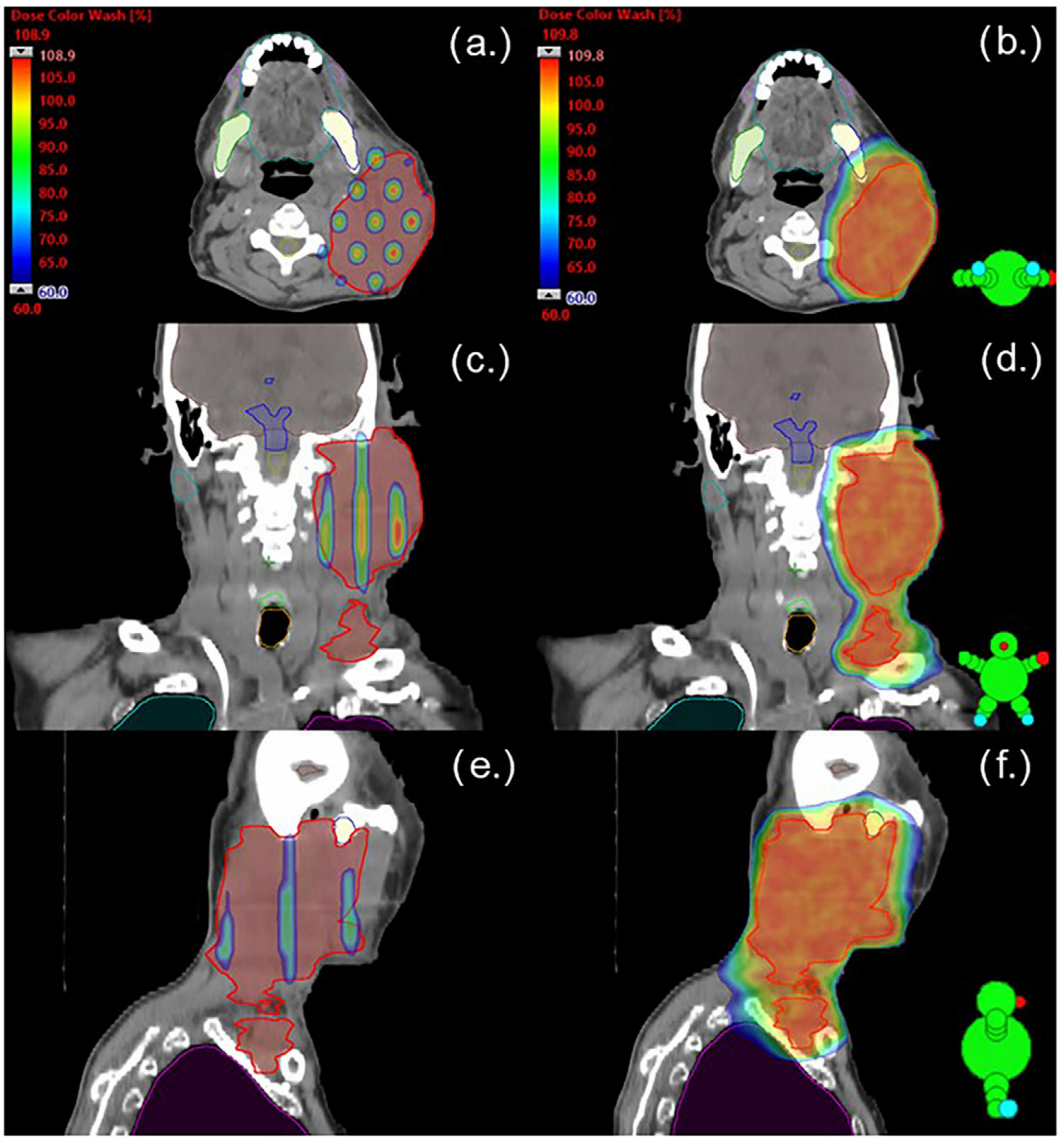
Demonstrated is a clinical HN case with a left neck mass, prescribed with an initial course of SFRT (15 Gy/1fx) followed up with a curative course (30 Gy/5fx). Shown in (a), (c), and (e) are the axial, coronal, and sagittal views of the SFRT course with an isodose color wash from 60–108.9% of 15 Gy. Shown in (b), (d), and (f) are the axial, coronal, and sagittal views of the follow‐up treatment course with an isodose color wash from 60–109.8% of 30 Gy. Shown is the contoured GTV (red), brain (brown), brainstem (blue), left mandible (dark blue), right mandible (green), oral cavity (light blue), trachea (light brown), lips (purple), larynx (light green), right parotid (cyan), and spinal cord (yellow).

### 3.3 Dosimetric reporting

The extracted dose metrics from the SFRT plan alone for all 137 treated tumors are shown in Table 2. Reported are the median values, as the dose metric results are not normally distributed in our cohort. The 3D MLC‐crossfire technique achieved median values of D5%, D10%, D50%, Dmean, D90%, D95%, EUD, and PVDR of 13.24 Gy, 11.80 Gy, 6.90 Gy, 7.27 Gy, 3.52 Gy, 2.38 Gy, 5.26 Gy, and 3.35, respectively. It should be noted that these values are higher than reported from another institution by Owen et al. ^29^ in which the majority of SFRT cases are prescribed at a higher prescription (20 Gy) and are planned mostly using lattice VMAT. These increases in tumor dose are seen with D10% (11.80 Gy vs 8.8 Gy), D90% (3.52 vs 2.1 Gy), mean dose (7.27 vs 5.1 Gy), and EUD (5.26 vs 3.6 Gy). However, the one downside is that our institutions reported PVDR is lower at 3.35 compared to that reported by Owen et al. ^29^ at 4.4.

**TABLE 2:** Extracted dose metrics from the SFRT plans alone from 137 treated tumors. Reported is the median value (range).

Dose metric	Median (Minimum—Maximum)
D5% (Gy)	13.24 (8.33–17.10)
D10% (Gy)	11.80 (6.99–14.86)
D50% (Gy)	6.89 (1.57–9.55)
Dmean (Gy)	7.27 (2.76–9.83)
D90% (Gy)	3.52 (0.06–6.73)
D95% (Gy)	2.38 (0.04–5.86)
PVDR = D10%÷D90%	3.35 (1.86–66.46)
EUD (Gy)	5.26 (1.74–8.03)

Also extracted were the BED metrics from the SFRT plan plus follow‐up combination treatments. BED metrics were also extracted for tumors that were treated with only SFRT. Table 3 shows the resulting extracted BED metrics. On average, these tumors were treated to a mean BED of 54.63 Gy. The range of extracted metrics is broad because the tumors were treated with diverse post‐SFRT dose prescriptions.

**TABLE 3:** Extracted dose metrics from both the SFRT and follow‐up plans if prescribed from a total of 137 treated tumors. Due to the highly heterogenous distribution of the SFRT plan and the differences in fractionation, a converted s‐BED was used to extract the BED dose metrics. Reported is the median value (range).

BED Metric	Median (Minimum—Maximum)
BED5% (Gy)	71.53 (22.06–170.20)
BED10% (Gy)	66.90 (18.00–159.43)
BED50% (Gy)	52.83 (8.73–105.01)
BEDmean (Gy)	54.63 (9.76–106.90)
BED90% (Gy)	46.15 (2.63–95.06)
BED95% (Gy)	44.43 (1.92–93.54)

Lastly, Table 4 provides the median maximum EQD2 to contoured OARs across our SFRT cohort. This table includes OARs that were contoured at least 30 times across the cohort. Also shown in the table are the ranges of values achieved in these clinical cases.

**TABLE 4:** Reported average Maximum EQD2 for adjacent OARs across the SFRT patient cohort. Reported is the median and range. Additionally reported is the number of instances the OAR was contoured in our cohort.

OAR	Median EQD2 (Maximum, Gy)	Dose range (Gy)	# Times contoured
Bladder	26.47	(0.00–102.24)	42
Bowel	45.87	(0.04–105.52)	57
Brain	34.65	(0.04–91.33)	43
Brainstem	16.08	(0.01–60.23)	41
Esophagus	40.39	(0.06–88.06)	83
Heart	1.41	(0.01–85.55)	56
Larynx	47.53	(0.17–113.70)	49
Lens	0.98	(0.01–74.81)	36
Liver	3.98	(0.05–114.28)	39
Lung	47.97	(0.03–123.18)	86
Mandible	56.59	(0.03–137.05)	59
Optic Chiasm	1.21	(0.01–23.00)	34
Parotid	65.23	(0.02–150.98)	45
Rectum	31.38	(0.09–73.18)	39
Skin	60.37	(16.04–127.51)	57
Spinal Cord	18.36	(0.00–47.29)	113
Stomach	2.44	(0.10–47.54)	33

### 3.4 Outcome reporting

Of the 137 lesions treated in the cohort, 98 were assessed for tumor control using the RECIST criteria. A total of 75 tumors (76.5%) were determined to be either stable disease, partial response, or a complete response, while 23 tumors (23.5%) were determined to be progressive disease. A total of 82 patients were assessed for pain relief, with 59 patients (72.0%) reporting improved pain relief.

Across our cohort, 95 patients were evaluated for toxicities. Out of these 95 patients, 65 (68.4%) reported no toxicities, while 7 (7.4%) reported a grade 3+ toxicity. Of the 30 complications, 11 were skin‐related, and 5 were xerostomia‐related. For the skin‐related toxicities, three of which were grade 1, three were grade 2, four were grade 3, and one reported patient experienced a grade 4 wound complication.

Lastly, OS follow‐up data were available for 117 patients. Across the entire cohort, 6‐month OS was 51.8%, 1‐year OS was 36.3%, and 18‐month OS was 30.3%. Figure 3 displays the Kaplan‐Meier curves stratified by post‐SFRT treatment intent. Across the whole cohort, the median OS time was 6.27 months. There was a statistically significant difference in OS between the two groups (*p* = 0.002). For the curative cohort, the 6‐month, 12‐month, and 18‐month OS rates were 65.4%, 55.0%, and 50.4%, respectively. For the palliative cohort, the 6‐month, 1‐year, and 18‐month OS rates were 41.6%, 22.4%, and 16.8%, respectively.

**FIGURE 3:**
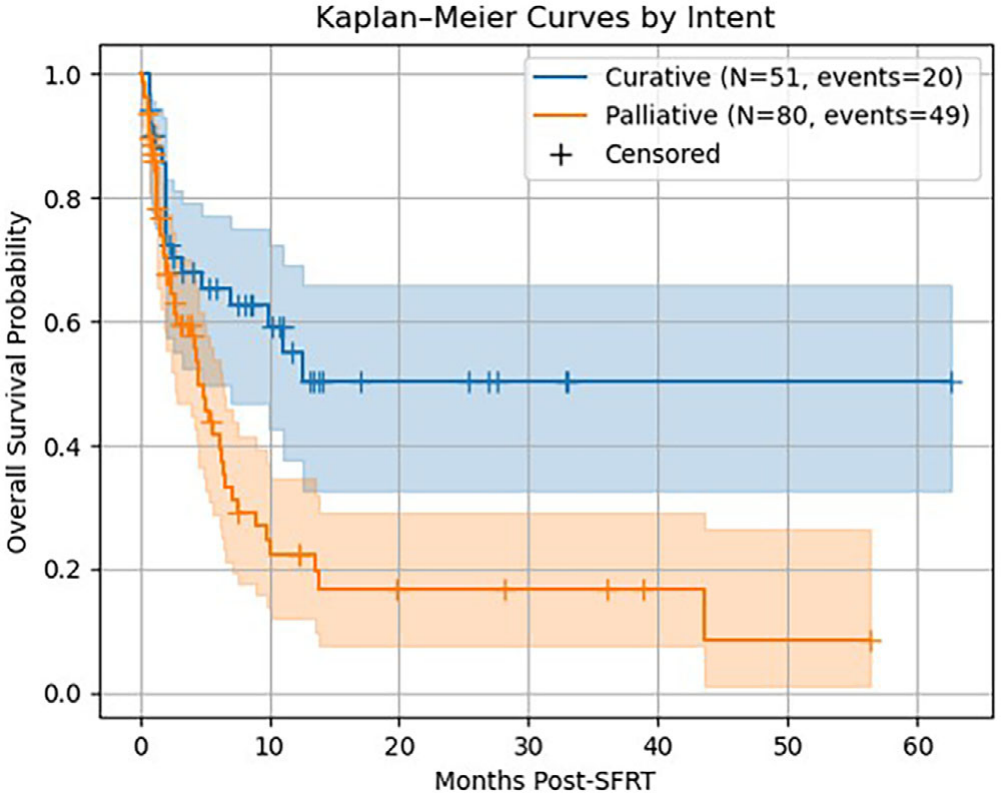
The Kaplan‐Meier curves for our SFRT patient cohort, stratified by treatment intent. Patients not receiving follow‐up treatment were included in the palliative group. Blue represents the curative cohort, while orange represents the palliative cohort. The shaded bands represent the 95% confidence interval.

### 3.5 Outcome modeling

A multivariable logistic regression model was used to analyze patient characteristics and dosimetric parameters for either the combined treatments or the SFRT course alone in relation to the outcome data. So for TCP and pain relief each, two multivariable models were created. Figure 4 shows the ROC curve derived from all four models. The apparent AUC from the TCP logistic models was 0.743 for the cumulative BED metrics and 0.727 for the SFRT dose metrics, indicating these models predict tumor local control fairly well when using either the cumulative BED metrics or the SFRT dose metrics. However, none of the metrics used in the models were statistically significant. The calculated optimism‐corrected AUC for the TCP model was 0.607 for the cumulative BED metrics and 0.656 for the SFRT dose metrics. This decrease in AUC indicate that model has been overfitted to the original data set, and may not have the same predictive power in different data sets. The same pattern was observed with the pain relief model, which showed acceptable AUC scores of 0.690 for cumulative BED metrics and 0.800 for SFRT dose metrics. Again, none of the dose metrics showed a statistically significant correlation with outcomes (*p* > 0.05). The calculated optimism‐corrected AUCs for the pain relief model was 0.536 for the cumulative BED metrics and 0.724 for the SFRT dose metrics, again showing the effects of our models overfitting to our data. Notably, in the TCP model, AUC scores were similar, but in the pain relief model, the model using the SFRT dose metric had a much higher AUC.

**FIGURE 4:**
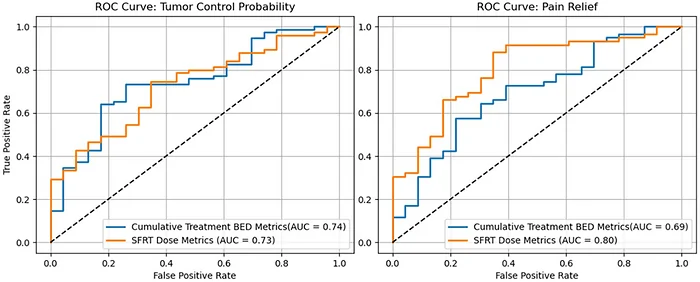
Shown are the resulting ROC curves from the multivariable logistic models to predict either tumor control (left) or pain relief (right). For each outcome prediction, two models were produced: one fitted to the cumulative treatment's BED metrics (blue) and one fitted to the SFRT plan's dose metrics (orange). Displayed AUCs were the apparent AUC from the original dataset.

Using univariable logistic regression, skin toxicities were modeled using max EQD2 to the skin, while xerostomia was modeled using both mean EQD2 and max EQD2 to the parotids. The maximum EQD2 to the skin was statistically significantly correlated with skin toxicities (*p* = 0.022), with an odds ratio of 1.03 (95% CI: 1.00–1.07). The mean EQD2 to the parotids was not statistically significantly correlated with xerostomia (*p* = 0.136). However, max EQD2 to the parotids was statistically significantly correlated (*p* = 0.038) with an odds ratio of 1.03 (1.00–1.07). Figure 5 shows the resulting NTCP logistic regression model curves for both skin toxicities and xerostomia.

**FIGURE 5:**
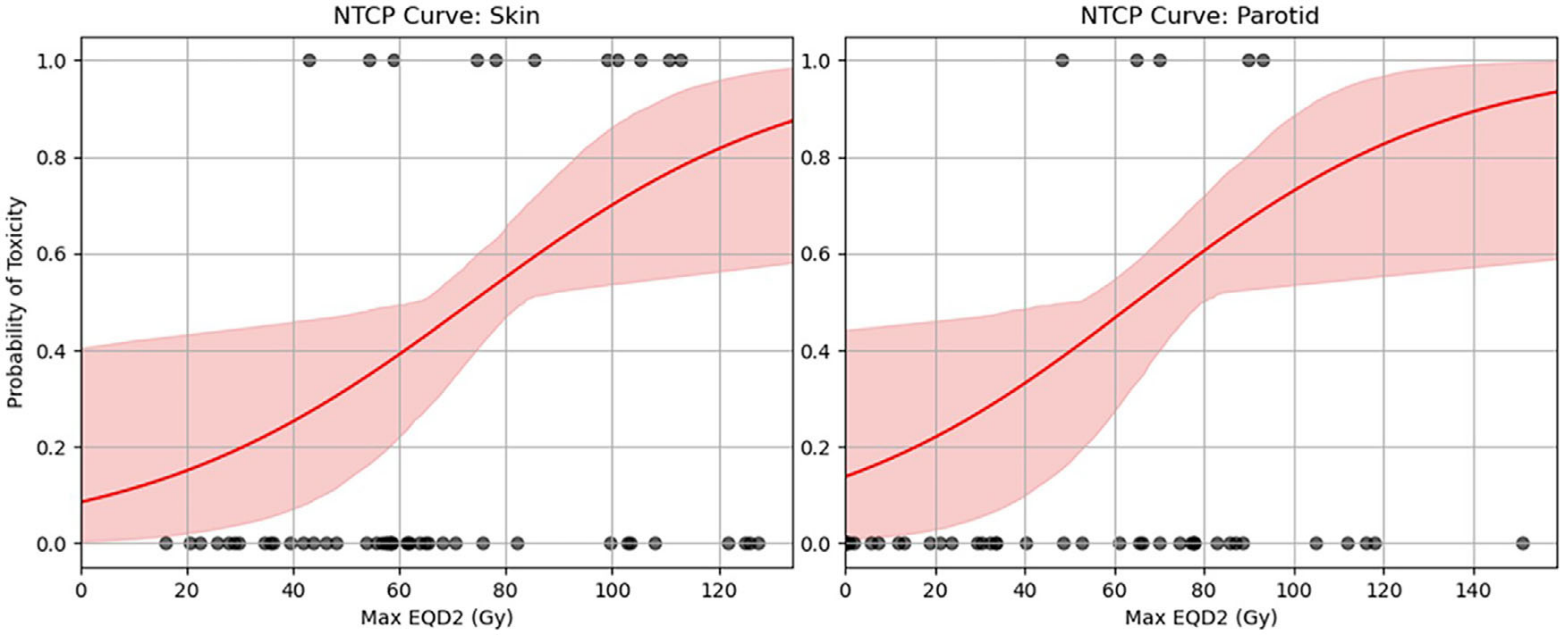
The resulting univariable logistic regression models for skin toxicity (left) and xerostomia (right) for SFRT patients. Black dots represent the individual data points, the red line represents the logistic model, and the light red band represents the 95% confidence interval.

Lastly, OS was modeled using a univariable Cox hazard model. The model was fitted with the cumulative treatment's BED metrics, the SFRT plan metrics, and with patient characteristics (treatment intent, treatment site, tumor volume). None of the patient characteristics were statistically significantly correlated with OS. The same was observed when analyzing only the SFRT plan's dose metrics, except for EUD (Table 5). An increase in EUD from the SFRT plan was statistically significantly correlated with a longer OS. Using BED metrics, we found that all dose metrics were statistically significantly correlated with OS. As shown in Table 6, all increases in these BED metrics are associated with improved OS, with hazard ratios (HRs) less than 1.

**TABLE 5:** Cox Hazards models’ analysis of SFRT plans doses metrics associated with OS. Hazard ratios (HR) with 95% confidence intervals (CI) and corresponding *p*‐values are shown for each variable.

Plan dose metric	HR	95% CI	*P*‐Value
D5% (per 1 Gy)	0.963	(0.817–1.14)	0.658
D10% (per 1 Gy)	0.956	(0.810–1.13)	0.593
D50% (per 1 Gy)	0.949	(0.813–1.11)	0.510
Dmean (per 1 Gy)	0.947	(0.804–1.12)	0.514
D90% (per 1 Gy)	0.952	(0.841–1.08)	0.443
D95% (per 1 Gy)	0.962	(0.838–1.11)	0.587
EUD (per 1 Gy)	0.829	(0.706–0.975)	**0.023**
PVDR (per 1)	1.01	(0.994–1.02)	0.294

**TABLE 6:** Cox hazards models analysis of cumulative treatment's BED metrics associated with OS. Hazard ratios (HR) with 95% confidence intervals (CI) and corresponding *p*‐values are shown for each variable. Bolded *p*‐values represent *p* < 0.05.

Plan dose metric	HR	95% CI	*P*‐Value
BED5% (per 1 Gy)	0.980	(0.973–0.993)	**0.001**
BED10% (per 1 Gy)	0.979	(0.973–0.993)	**0.001**
BED50% (per 1 Gy)	0.981	(0.975–0.996)	**0.006**
BEDmean (per 1 Gy)	0.981	(0.975–0.995)	**0.005**
BED90% (per 1 Gy)	0.985	(0.980–0.998)	**0.023**
BED95% (per 1 Gy)	0.986	(0.980–0.999)	**0.025**

## 4 DISCUSSION

In this study, we report our institution's clinical outcomes and experience with using SFRT planned using the 3D MLC‐Crossfire technique ^6^, ^21^, ^22^. Our patient cohort consisted of 137 lesions across 131 patients with a median follow‐up interval of 3.23 months (max: 64 months) and with a median tumor volume of 477.55 cc. Most SFRT courses were prescribed at a nominal dose of 15 Gy in 1 fraction (85.4%). The majority of tumors (92.0%) received an additional course of radiation after SFRT, with more than half receiving a palliative course (59.5%). This study extracted and analyzed SFRT dose metrics, as well as BED and EQD2 metrics, from the cumulative treatment (SFRT plus follow‐up courses of combination RT) using an s‐BED methodology ^25^.

Comparisons of our SFRT dose metrics with those from other reported institutional studies are difficult due to differences in prescribing practices. The LITE‐SABR trial reported by Washington University, St. Louis ^30^, ^31^ and a study by Studer et al. ^32^ used a prescription of 20 Gy in 5 fractions. In contrast, a study by Owen et al. ^29^ primarily used a prescription of 20 Gy in 1 fraction. Another study by Iori et al. ^33^ used a prescription of 20 Gy in 5 fractions, while a study by Xu et al. ^34^ used a prescription of 12 Gy peak dose per fraction delivered over 2–3 fractions. The prescription our clinic uses more closely aligns with studies performed in the late 1990s and early 2000s ^1^, ^2^, ^3^, ^4^, ^5^, ^35^. In comparison with Owen et al. ^29^, who reported SFRT dose metrics, our technique resulted in higher median D10%, D90%, mean dose, and EUD to GTV. However, the drawback is that our reported PVDR was, on average, slightly lower, likely due to differences in planning techniques. Their study primarily used an inverse‐optimized VMAT plan for SFRT, which provides better control of the dose distribution around the vertex, resulting in a slightly larger PVDR than with forward planning techniques ^36^. Lastly, our study also reported GTV BED metrics from cumulative radiation therapy treatments, which have not been reported before.

Assessment of patient outcomes included tumor local control, pain relief, toxicity concerns, and OS. Even though using a simple 3D MLC‐based method, our studies reported tumor local control and pain relief rates that closely align with results from other institutions ^29^, ^31^, ^32^, ^33^, ^34^. Additionally, our reported toxicity rates were also found to be similar to Owen et al. ^29^, with our study seeing a grade 3+ toxicity rate of less than 7.4% with their study reporting 5.1%. We also found that the OS rates at 6 months (57% vs 51.8%), 12 months (37% vs 36.3%), and 18 months (29% vs 30.3%) were very similar between our studies. However, our study's cohort showed a statistically significant difference in OS between the palliative and curative cohorts.

Our study also modeled tumor control, pain relief, toxicities, and OS using either a multivariable logistic regression model, an univariable logistic regression model, or a univariable Cox Hazard model. Regarding the TCP and pain relief multivariable logistic regression models, we found that none of the extracted dosimetric parameters or patient characteristics were statistically significantly correlated with treatment outcomes during clinical follow‐up. However, the models may have some predictive potential. We found that, for predicting pain relief, the SFRT dose metrics were more predictive than the cumulative treatment's BED metrics, as indicated by the higher apparent AUC value of 0.80 shown in Figure 4. Additionally, we found little difference between using either the SFRT dose metrics or the cumulative treatment BED metrics for modeling TCP, as indicated by the apparent AUC values in Figure 4. Because none of the metrics were statistically significant, this may indicate that currently used metrics for evaluating SFRT plans may be inadequate. The relatively limited sample size may have attributed to the lack of statistical power in distinguishing metrics that may have a statistically significant correlation with the outcomes. Future work is underway to investigate potential new metrics that may be statistically predictive, but also to include multi‐center patient cohorts. For NTCP modeling, we investigated only skin toxicities or xerostomia, as these were the only two toxicities that occurred ≥ 5 times in our SFRT cohort. We found that maximum EQD2 to the skin and parotid glands were statistically significantly correlated with skin toxicities and xerostomia, respectively, as demonstrated with their p‐values. However, the associated odds ratio and confidence intervals indicate that the increase in NTCP with an increase in maximum EQD2 is small, as we reported an odds ratio of 1.03 for both skin‐related toxicities and xerostomia. The odds ratio results may suggest a limited clinical effect. Interestingly, the mean dose to the parotids is a metric often evaluated in treatment planning for xerostomia, but the mean EQD2 to the parotids was not statistically significantly correlated with the patient's dry mouth.

Lastly, we modeled OS using an univariable Cox Proportional Hazards model and estimated OS using the Kaplan‐Meier method. We found a statistically significant difference in OS between the palliative and curative SFRT cohorts. We also saw that the cumulative treatment's BED metrics were statistically significantly correlated with OS; however, the SFRT metrics were not. The only exception was the EUD from the SFRT, which showed a statistically significant correlation with OS time. From this, it's not the SFRT plan doses, but rather the follow‐up courses that are more important for OS rates. But this may be due to differences in curative and palliative care post‐SFRT, as we see a difference in OS between the cohorts.

The main limitation of our study is the high heterogeneity of our patient population. Our SFRT cohort comprises a wide range of histopathologies, treatment sites, tumor sizes, and OAR proximity. Additionally, we observe heterogeneity in prescribing both the SFRT and follow‐up courses, driven by tumor type, size, and location, as shown in Table 1. Another limitation is the small patient cohort, which limits modeling outcomes with many dosimetric variables. This limited number of outcomes per variable investigated increases the risk of overfitting and limits the generalizability of our predictive models. This may be a potential reason why our multivariable logistic regression models lacked a statistically significant metric association with outcomes. Future work should include multi‐institutional studies to provide external validation of our model's findings, including prospective clinical trials of SFRT with combination therapy.

A limitation with our TCP, pain relief, and OS model is that the metrics used as parameters may have a high correlation with each other while being incorporated into these models simultaneously. This work didn't evaluate the multicollinearity of these metrics when developing the model, which may contribute to instability in the model's coefficients and limit the statistical ability to evaluate the contributions of each metric's ability to predict outcomes. Additionally, with the limited number of events in our cohort, this limitation makes our models at risk for overfitting and may lead to overestimating the predictive power of our models. This effect is seen with the difference in apparent and optimism‐corrected AUC in the TCP and pain relief model. These differences in AUCs indicate that our models may not be generalizable. Because of this, the results of these outcomes models presented should be interpreted with care. Furthermore, the results of the optimism‐corrected AUCs further strengthen the need for external validation data sets.

Another major limitation of our models is that we utilized BED and EQD2 metrics calculated using the linear‐quadratic model. It has been established in previous literature that the linear‐quadratic model breaks down at higher dose per fraction due to the additional cell killing from indirect cell kill mechanisms ^37^. It has also been clinically shown that the linear‐quadratic model underestimates tumor control for high‐dose per fraction ^38^. The main issue is that there hasn't been a clinically accepted model for calculating BED and EQD2 for these higher dose per fraction treatment techniques. More specifically, there isn't a clinically accepted cell‐survival model for SFRT treatments. Future research would include incorporating other cell‐survival models to re‐derive the extracted dose metrics to feed into our models.

A major hurdle with this study is differentiating the effects of tumor control, pain relief, and OS between the SFRT course and from the follow‐up courses. We did observe a statistical difference between OS rates between patients who received palliative versus definitive follow up course of radiation. But for local tumor control and pain relief, it is difficult attributing how much contribution each course has with the resulting patient outcomes. This consideration should be kept when interpreting these results.

## 5 CONCLUSION

We reported our single institution's clinical experience with SFRT using a 3D‐MLC‐based crossfire technique, with patients reporting follow‐up results. We reported clinical benefits of a simple MLC‐based 3D method for tumor local control and pain relief, consistent with other institutions using advanced SFRT methods, including inversely optimized VMAT plans. We observed comparable OS rates to those of other institutions, with minimal radiation‐induced toxicity. We also modeled our outcomes using dosimetric parameters from the SFRT plans plus follow‐up combination treatments. We found that the current metrics recommended by AAPM and RSS SFRT working groups were not predictive of the TCP or pain relief. We also found that in this SFRT cohort, the OS rate was mainly affected by the combination of follow‐up radiation therapy. Further collection of multi‐institutional data is needed to externally validate our models and findings.

## AUTHOR CONTRIBUTIONS

Damodar Pokhrel, PhD, and Josh Misa, PhD designed the 3D MLC‐based SFRT treatment method, developed MLC fitting algorithm and generated clinical SFRT plans. “Damodar Pokhrel, PhD, Josh Misa, PhD and Jovan Pierre Charles, MD, collected and analyzed the data”. Jovan Pierre‐Charles, MD, and William St. Clair, MD, PhD provided clinical follow up radiation oncology clinical expertise. Josh Misa, MS and Damodar Pokhrel, PhD drafted the preliminary manuscript. All co‐authors revised, edited, and approved the final document for submission.

## CONFLICT OF INTEREST STATEMENT

The authors declare no conflicts of interest.

## ETHICAL APPROVAL

This retrospective clinical improvement study was granted Exempt/Minimal Risk status by the University of Kentucky Institutional Review Board (IRB approval # 92667). All research was conducted in strict accordance with IRB regulations and institutional ethical guidelines.

## Data Availability

No data available on request due to privacy/ethical restrictions.
